# Trajectories of fluid management after the initiation of renal replacement therapy in critically ill patients: a secondary analysis of the STARRT-AKI trial

**DOI:** 10.1186/s13054-025-05447-y

**Published:** 2025-05-27

**Authors:** William Beaubien-Souligny, Ehsan Gamarian, Jean-Maxime Côté, Javier A. Neyra, Frederic Baroz, Neill K. J. Adhikari, Kevin Thorpe, Sean M. Bagshaw, Ron Wald

**Affiliations:** 1 Division of Nephrology, Centre Hospitalier de l’Université de Montréal, Montreal, Canada; 2 Centre de Recherche du Centre Hospitalier de l’Université de Montréal, Montreal, Canada; 3 Applied Health Research Centre (AHRC), Toronto, Canada; 4 Division of Nephrology, Department of Medicine, University of Alabama at Birmingham, Birmingham, AL USA; 5 McGill University, Montreal, Canada; 6 Department of Critical Care Medicine, Sunnybrook Health Sciences Centre, and Interdepartmental Division of Critical Care Medicine, University of Toronto, Toronto, Canada; 7 Dalla Lana School of Public Health, University of Toronto, Toronto, Canada; 8 Department of Critical Care Medicine, Faculty of Medicine and Dentistry, University of Albert and Alberta Health Services, Edmonton, Canada; 9 Division of Nephrology, St. Michael’s Hospital, Toronto, Canada; 10 Department of Nephrology and Hypertension, Tel Aviv Medical Center, Tel Aviv, Israel

**Keywords:** Renal replacement therapy, Acute kidney injury, Fluid balance, Ultrafiltration

## Abstract

**Background:**

Fluid management is an essential component of renal replacement therapy (RRT) in critically ill patients. Both a positive cumulative fluid balance (CFB) and a high net ultrafiltration (NUF) rate have been reported to be associated with adverse outcomes in epidemiological studies, although the overall trajectory of fluid balance after RRT initiation is not well-described. We aimed to characterize trajectories of fluid management parameters during RRT and analyse the effect of CFB/NUF on outcomes as a trajectory rather than single or aggregated time points over the first week after initiation of RRT.

**Methods:**

This is a secondary analysis using fluid balance data focusing on individuals enrolled in the standard-strategy arm of the STARRT-AKI trial who initiated RRT. Cumulative fluid balance (CFB) following RRT initiation and daily net ultrafiltration (NUF) adjusted for body weight during the first 7 days after initiation of RRT were the main independent exposures. We modeled the trajectory of fluid parameters using spline functions and used latent trajectory analysis methods to identify predominant trajectories to compare patients’ characteristics and outcomes. We employed logistic regression and multivariable joint longitudinal models to compare the odds and determine the time-dependent association between fluid parameters (CFB and NUF) and 90-day mortality across and within the trajectory classes identified.

**Results:**

We included 855 patients in the primary analysis. After excluding erroneous fluid balance data, we identified two distinct CFB/NUF trajectories. Class A (82.8%) was characterized by a slight increase in CFB and low/stable NUF during the week following RRT initiation while class B (17.2%) was characterized by an increasingly negative CFB with initially higher daily NUF during the first 4 days followed by a stabilization after day 4. In an adjusted analysis, individuals classified in class B were at lower risk for 90-day mortality (aOR: 0.48 CI 0.32; 0.70) *p* < 0.001) compared to class A. Time-dependent analysis revealed higher CFB was associated with mortality only in those with a class A trajectory (aHR 1.29, 95% CI 1.03–1.55, *p* = 0.03).

**Conclusions:**

Distinct CFB/NUF trajectories convey prognostic information beyond single-day fluid balance or NUF values and should be considered when formulating or interpreting fluid management strategies.

**Supplementary Information:**

The online version contains supplementary material available at 10.1186/s13054-025-05447-y.

## Introduction

Fluid removal is an essential component of renal replacement therapy (RRT) in critically ill patients with severe acute kidney injury (AKI). In most cases, significant fluid accumulation has occurred prior to RRT initiation, since fluid administered through resuscitative efforts and intravenous medications often cannot be eliminated due to decreased or absent urine output. Beyond the immediate danger of respiratory compromise from pulmonary congestion, it has been hypothesized than fluid accumulation may mediate adverse outcomes through the congestion of peripheral organs [1]. Accordingly, fluid accumulation at the start of RRT has been repeatedly reported to be a risk factor for mortality [2–4], while the successful induction of a negative fluid balance during the first days of RRT or overall fluid removal goals is associated with improved prognosis [5, 6]. However, it is still uncertain whether using a high net ultrafiltration (NUF) rate to reach this objective is safe, since mechanical fluid removal may result in hypotension, increased vasopressor doses, and sub-clinical organ ischemia [7, 8]. Based on several observational cohorts, it has been suggested that a high NUF rate (> 1.75 mL/kg/h) among patients receiving continuous RRT is associated with adverse clinical outcomes including early mortality [9–11] and RRT dependence [12], and this association appears to be only partially attenuated by the degree of fluid accumulation but still susceptible to indication bias [13].

Since it is well established that fluid removal is a dynamic parameter that is adjusted over time after initiation of RRT, we hypothesized that associations between NUF intensity or CFB after RRT initiation and patient outcomes are incompletely described by previous efforts using single time points or aggregated data. In this secondary analysis of data collected during the multinational *Standard versus Accelerated Initiation of Renal-Replacement Therapy in Acute Kidney Injury* (STARRT-AKI) trial [14], we aimed to determine the most common trajectories of NUF and cumulative fluid balance (CFB) over time after the initiation of RRT. We then used joint longitudinal modeling and latent trajectory analysis to identify dominant trajectories of CFB and NUF and describe their association with patients’ characteristics and outcomes.

## Methods

### Design and participants

This is a secondary analysis of data from participants in the Standard versus Accelerated Initiation of Renal-Replacement Therapy in Acute Kidney Injury (STARRT-AKI) trial who initiated renal replacement therapy (RRT) during the first 14 days after enrolment [14]. To be eligible for the STARRT-AKI trial, patients 18 years or older had to be admitted to an intensive care unit (ICU) with severe AKI categorized as stage 2 or 3 of the *Kidney Disease: Improving Global Outcomes* (KDIGO) classification. Importantly, eligibility was predicated on the patient not having an urgent reason for RRT initiation, including severe volume overload, or a compelling reason for RRT deferral. The complete inclusion and exclusion criteria have been described [14]. Initiation of RRT in the STARRT-AKI trial was performed within 12 h of enrolment in the accelerated arm. In the standard arm, RRT was to be initiated if urgent criteria emerged, or at the discretion of the attending clinical care team after 72 h. However, RRT initiation could always be initiated or deferred as per clinician judgement.

In this secondary analysis, all participants who initiated RRT in the standard arm of the trial within 14 days of enrolment were included. We chose to restrict the analysis to participants in the standard arm since the timing of initiation of RRT is more representative of usual care compared with the accelerated arm, in which the protocol mandated RRT initiation and conferred a modest attenuation of CFB [15]. Cohort assembly is depicted in Figure S1 of the supplementary material. The primary analytical cohort for this analysis consisted of 855 patients. In sensitivity analyses, we also studied an alternative cohort of 1347 patients who were enrolled in the accelerated arm of the trial.

### Definition of exposure variables and outcomes

The primary exposure variables were cumulative fluid balance (CFB) and daily net ultrafiltration (NUF) since the day of RRT initiation, expressed in mL/kg based on ICU admission weight, through day 7 after RRT initiation. CFB was calculated from daily total fluid balance data collected daily for 14 days after enrolment in the trial and included all the intake and output (from all sources) without correction for insensible losses. NUF was defined as net volume removed by the RRT machine. The primary outcome was mortality up to 90 days after enrolment. The Sequential Organ Failure Assessment (SOFA) score was determined based of the actual capacity of the patient [16] (see supplementary material).

### Modelling

The initial aim was to determine if CFB and NUF after RRT initiation were associated with mortality. However, preliminary steps involved in the verification of assumptions regarding the model (see joint modelling methods below) revealed evidence for heterogeneity of CFB and NUF (SD > mean) (Table S1 of supplementary material). Furthermore, the effect estimates of the association between CFB and 90-day mortality was not constant over the duration of the observation period (Figure S2 of supplementary material). We therefore hypothesized that the observed heterogeneity could imply latent trajectories that may be better characterized through the use of methods aiming to identify these classes [17].

To capture potential heterogeneity in trajectory patterns within the cohort, we utilized a longitudinal latent class mixed modeling approach. This method is a variant of latent class analysis allowing us to produce data-driven clusters based on trajectories of continuous indicators (CFB and NUF) and reduce the within-group variance and non-linearity of hazard over time. We used restricted cubic splines with three knots in both random and fixed parts of the model to flexibly model the nonlinear trajectories of CFB and NUF over time and to allow for further flexibility and heterogeneity in trajectory shapes across latent classes. Random effects for both fixed and random parts of the model were included to account for individual-specific variation in baseline levels and trajectory shapes. To capture potential subgroups with distinct trajectory profiles, we allowed for two, three and four latent classes in the models. We selected the final number of classes based on the Bayesian information criterion (BIC), entropy (> 0.8), the prevalence of each class and clinical interpretability [17].

To compare 90-day mortality between the two latent classes, we used a multivariable logistic regression model using the latent trajectory membership as the predictor with adjustments for potential confounders including age, comorbidities including diabetes, chronic kidney disease and heart failure, as well as information related to current illness including the presence of sepsis, the receipt of cardiopulmonary bypass for cardiothoracic surgery in the prior 7 days before enrolment, and CFB from ICU admission to initiation of RRT. Age was standardized across the entire cohort and fluid balance was scaled by patients’ weights and converted to liter per kg of measured body weight. In a sensitivity analysis, we added the probability of membership to the most prevalent class as an adjustment variable.

We also employed a joint modelling approach to investigate the relationship between 90-day survival and longitudinal trajectories of CFB/NUF across and within each of the identified latent classes [18]. For the longitudinal component, we utilized a multivariable generalized linear mixed-effects model. We allowed for flexible and simultaneous non-linear modelling of both CFB and an NUF trajectories over time since RRT initiation up to 7 days. We also accounted for within-subject correlations through random intercepts and slopes at the subject level. Survival outcome was analyzed using a Cox proportional hazards model and adjusted for aforementioned potential confounders. Results are presented as Hazard Ratio (HR) with 95% confidence intervals (CI).

We performed additional sensitivity analyses. First, we assessed whether an interaction was present between CFB before RRT initiation and the studied classes in regard to the association with 90-day mortality. Second, we reproduced the aforementioned steps using data available from the participants enrolled in the accelerated arm of the trial in order to determine if similar trajectories would be found and whether they would also be associated with 90-day mortality.

Analysis was done in R (Version 4.2.2). The package lcmms [18] and JMbayes [19] were used for latent class analysis and jointed longitudinal survival analysis.

## Results

### Patient characteristics

Of the 903 participants who initiated RRT in the standard arm of the STARRT-AKI trial, we included 855 patients in the analytical cohort after excluding patients with missing fluid balance data (Supplementary material, Figure S1). Characteristics of included patients are presented in Table 1. The mean time from ICU admission to RRT initiation was 134 ± 345 h and the mean CFB from ICU admission to RRT initiation was 57 ± 72 mL/kg. Most patients were receiving mechanical ventilation (83%) and vasopressor support (62%). At 90-days after enrolment, 51% of participants had died.

**Table 1:** Characteristics of patients included in the analytical cohort

Characteristic	N = 855
*Baseline characteristics*	
Age (Years)	64(13)
Body weight (Kg)	87(24)
Baseline GFR (mL/kg/1.73m2)	67(30)
Known chronic kidney disease (N (%))	366 (43%)
Known hypertension (N (%))	474 (55%)
Known diabetes (N (%))	271 (32%)
Known heart failure (N (%))	112 (13%)
Cardiopulmonary bypass (N (%))	66 (7.7%)
Sepsis (N (%))	515 (60%)
Aortic aneurysm repair (N (%))	45 (5.3%)
Other vascular surgery (N (%))	43 (5.0%)
Trauma (N (%))	27 (3.2%)
*Illness at RRT initiation*	
Time from ICU admission to RRT initiation (Hours)	134(345)
Cumulative Fluid Balance (mL/Kg)	57 (72)
24 h Urine Output prior to RRT initiation (mL/kg)	747(996)
Vasopressor support (N (%))	530 (62%)
SOFA score (points)	12.2(3.5)
- Respiratory component	2.16(1.10)
- Coagulation component	1.15(1.18)
- Liver component	0.87(1.17)
- Cardiovascular component	2.44(1.71)
- Central nervous system component	2.42(1.52)
- Renal component	3.22(0.88)
Mechanical ventilation (N (%))	713 (83%)
*Laboratory values at RRT initiation*	
Hemoglobin(g/dL)	9.27(1.80)
White blood cell count (× 10^9^/L)	18(15)
Platelets (× 10^9^/L)	160(116)
Serum bilirubin (mg/dL)	47(84)
Arterial pH	7.31(0.10)
Serum sodium (mmol/L)	138(7)
Serum creatinine (mg/dL)	4.85(2.10)
Serum potassium (mmol/L)	4.59(0.79)
Serum bicarbonate (mmol/L)	19.5(4.9)
Blood urea nitrogen (mg/dL)	30(24)
*Outcomes*	
RRT required at day 90 (N (%))	40 (4.7%)
Death at 90 days (N (%))	436 (51%)
Death between 1 and 7 days	188 (22%)
Death between 8 and 90 days*	248 (37%)
ICU death (N (%))	342 (40%)

eGFR, estimated glomerular filtration rate; ICU, intensive care unit; RRT, renal replacement therapy; SOFA, sequential organ failure assessment

*Among survivors at day 7 (N = 667). Data is presented in count (%) or mean (SD)

### Latent trajectories of NUF/CFB

We considered the potential presence of 2, 3 and 4 latent groups in the data. The model evaluation criteria are presented in Table 2. The model with four latent classes had the lowest BIC (-53,413). However, two of the latent classes had very similar trajectories and could be categorized as one larger group and the entropy parameter indicated worse classification. We proceeded with three latent classes (BIC of -53,300, resulting in good class separation (entropy of 0.846).

**Table 2:** Model evaluation criteria according to the number of latent classes

Number of classes	Bayesian information criteria (BIC)	Entropy	% class 1	% class 2	% class 3	% class 4
1 Class	− 52,609.47	1.000	100%			
2 Class	− 53,010.54	0.733	83.7%	16.3%		
3 Class	− 53,300.80	0.846	2.9%	83.7%	16.3%	
4 Class	− 53,413.44	0.696	2.7%	8.4%	55.1%	33.8%

Latent class analysis revealed three distinct trajectories for CFB and NUF. Patients in the first latent class (n = 25) had elevated CFB attaining implausible values of CFB (i.e. 1L/kg of CFB) which likely represented artefacts of data collection (Figure S3 of the supplementary material). Based on this analysis, patients in this latent class were considered to have unreliable data and were removed from further analysis. Baseline characteristics of the outlier latent class are presented in supplementary Table S2.

**Fig. 1:**
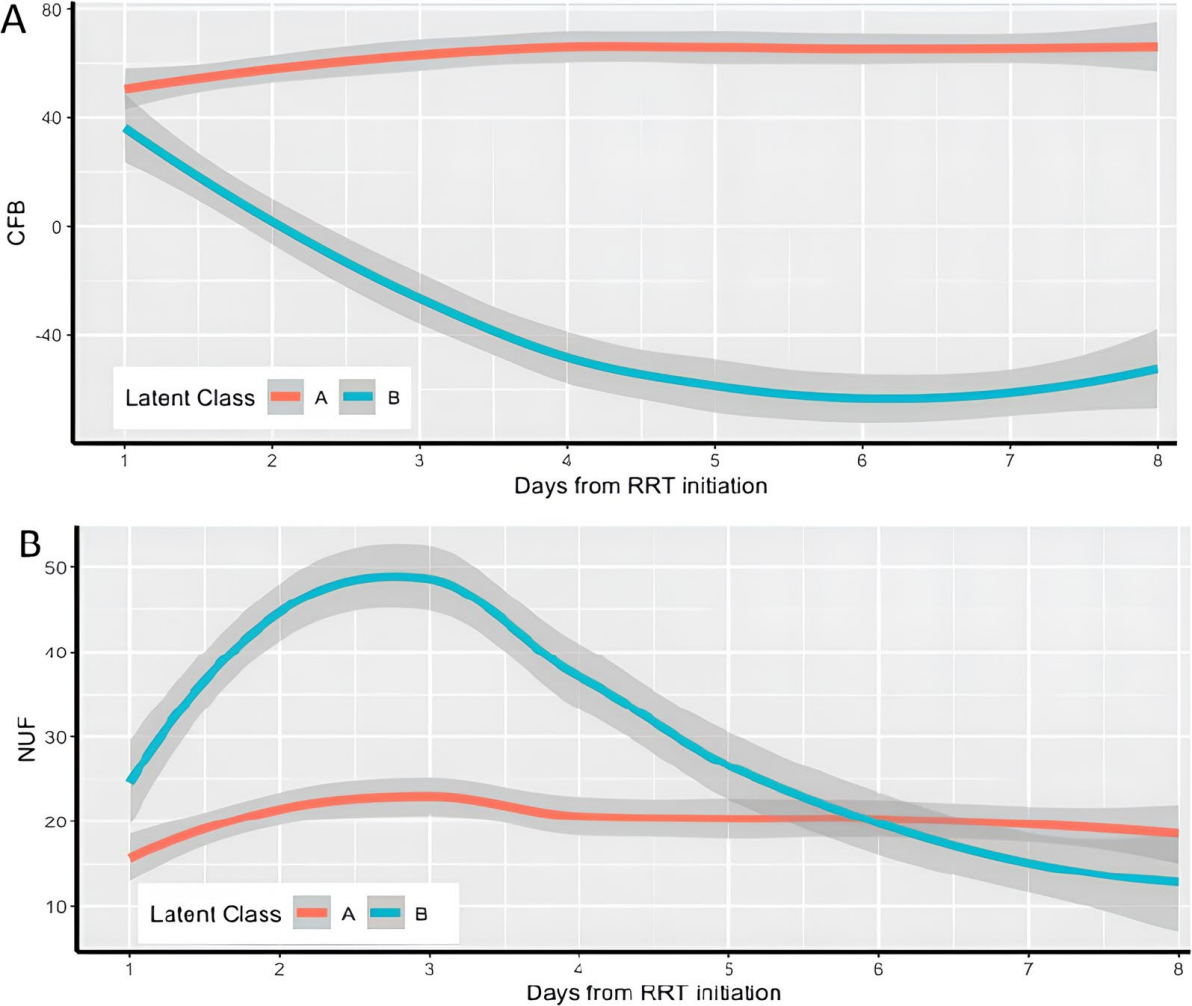
Predominant trajectories of **A** cumulative fluid balance (CFB) in mL/kg and **B** daily ultrafiltration rate (UF) in mL/kg/d identified using latent class analysis (Class A: 687 patients, Class B: 143 patients)

Patients in the second (Class A, n = 687) and third (Class B, n = 143) latent classes had a similar starting point in terms of CFB and NUF but subsequently differing trajectories shown in Fig. 1. Class A patients exhibited a slight increase in CFB over time after RRT initiation which stabilized after day 4 with a moderate and constant NUF intensity (equivalent to 1 mL/kg/h) throughout the observation period. Class B exhibited an increasingly negative CFB in the first 4 days followed by a plateau. The first 4 days were also characterized by higher NUF rates, with the maximal values observed at day 2–3 (equivalent to ≥ 1.75 mL/kg/h). Detailed fluid balance data is presented in Table 3.

**Table 3:** Fluid balance data after initiation of renal replacement therapy according to membership to classes of fluid management trajectories

Characteristic	Days after RRT initiation							
	Day 0 N = 687	Day 1 N = 656	Day 2 N = 609	Day 3 N = 562	Day 4 N = 515	Day 5 N = 476	Day 6 N = 436	Day 7 N = 407
Latent class A (N = 687)								
*Modality*								
Not on RRT	0 (0%)	108 (16.46%)	119 (19.54%)	181 (32.21%)	205 (39.81%)	176 (36.97%)	177 (40.60%)	167 (41.03%)
CRRT	469 (68.27%)	433 (66.01%)	374 (61.41%)	290 (51.60%)	219 (42.52%)	205 (43.07%)	175 (40.14%)	154 (37.84%)
IHD	191 (27.80%)	94 (14.33%)	97 (15.93%)	73 (12.99%)	76 (14.76%)	78 (16.39%)	73 (16.74%)	72 (17.69%)
PIRRT	27 (3.93%)	21 (3.20%)	19 (3.12%)	18 (3.20%)	15 (2.91%)	17 (3.57%)	11 (2.52%)	14 (3.44%)
UF achieved—(mL/Kg of BW)	8.63(21.77)	15.85(32.69)	13.47(30.37)	9.50(29.57)	6.05(30.77)	10.91(33.60)	5.10(33.20)	2.28(29.22)
Total daily FB—(mL/Kg of BW)	50.44(72.36)	8.13(23.77)	5.83(21.15)	4.09(22.65)	1.27(23.04)	-1.21(23.14)	0.21(22.49)	1.02(21.55)
Cumulative FB—(mL/Kg of BW)	50.44(72.36)	57.64(81.73)	63.24(90.20)	65.66(96.86)	66.55(109.14)	63.51(120.05)	65.44(133.15)	66.30(145.07)
*Latent class B (N = 143)*								
Modality								
Not on RRT	0 (0%)	7 (4.90%)	17 (12.06%)	29 (21.32%)	42 (33.33%)	48 (40.68%)	49 (45.37%)	45 (46.39%)
CRRT	118 (82.52%)	120 (83.92%)	112 (79.43%)	93 (68.38%)	74 (58.73%)	61 (51.69%)	45 (41.67%)	37 (38.14%)
IHD	20 (13.99%)	15 (10.49%)	11 (7.80%)	14 (10.29%)	9 (7.14%)	8 (6.78%)	9 (8.33%)	12 (12.37%)
PIRRT	5 (3.50%)	1 (0.70%)	1 (0.71%)	0 (0%)	1 (0.79%)	1 (0.85%)	5 (4.63%)	3 (3.09%)
UF achieved—(mL/Kg of BW)	16.17(25.80)	48.55(41.75)	41.88(38.13)	37.01(48.96)	18.21(43.84)	3.88(25.70)	2.18(27.86)	0.03(18.52)
Total daily FB—(mL/Kg of BW)	35.75(58.52)	− 32.58(23.46)	− 29.90(22.15)	− 23.32(26.24)	− 11.04(24.02)	− 0.53(24.12)	2.29(22.04)	8.35(19)
Cumulative FB—(mL/Kg of BW)	35.75(58.52)	3.16(61.06)	− 26.71(63.80)	− 48.73(73.11)	− 59.33(84.66)	− 60.56(96.16)	− 64.05(102.44)	− 51.36(109.57)

Data is presented in n (%); Mean(SD) or Median [IQR]

BW, body weight; CRRT, continuous renal replacement therapy; RRT, renal replacement therapy; IDH, intermittent hemodialysis; PIRRT, prolonged intermittent renal preplacement therapy

### Characteristics of patients according to latent trajectories of NUF/CFB

Characteristics of patients according to their membership in either class are presented in Table 4. Patients in class B had lower body weight (79 ± 19 vs. 89 ± 24 kg *p* < 0.001). At RRT initiation, patients in class B had a higher CFB since ICU admission (70 ± 80 vs. 51 ± 65, *p* = 0.007), a lower serum sodium (136 ± 7 vs. 138 ± 7, *p* = 0.002), and a slightly higher arterial high pH (7.34 ± 0.09 vs. 7.30 ± 0.10, *p* < 0.001). No differences were noted in terms of use of mechanical ventilation, or urine output before RRT initiation. Patients in class A were more likely to have criteria for sepsis in the previous 72 h (63% vs. 49%, *p* = 0.003). Although continuous RRT was chosen as the initial modality in the majority of patients in both classes, intermittent hemodialysis was chosen as the initial modality for a greater proportion of patients in class A (27.8% vs. 14.0%, *p* < 0.001) (Table 3).

**Table 4:** Patients characteristics according to their membership to identified latent classes

Characteristic	Class A N = 687	Class B N = 143	Mean/Risk Difference	95% CI	p-value
*Baseline characteristics*					
Age (Years)	65(13)	62(15)	2.6	-0.13, 5.3	0.06
Body weight (Kg)	89(24)	79(19)	10	6.7, 14	< 0.001
Baseline GFR (mL/kg/1.73m2)	67(30)	70(32)	-3.7	-9.4, 2.0	0.20
Known chronic kidney disease (N (%))	296 (43%)	57 (40%)	1.08	0.88, 1.36	0.50
Known hypertension (N (%))	390 (57%)	69 (48%)	1.18	0.99, 1.43	0.080
Known diabetes (N (%))	218 (32%)	45 (31%)	1.01	0.78, 1.34	> 0.90
Known heart failure (N (%))	90 (13%)	16 (11%)	1.17	0.73, 2.01	0.50
Cardipulmonary bypass (N (%))	50 (7.3%)	16 (11%)	0.65	0.39, 1.15	0.11
Sepsis (N (%))	430 (63%)	70 (49%)	1.28	1.08, 1.55	0.007
Aortic aneurysm repair (N (%))	35 (5.1%)	7 (4.9%)	1.04	0.50, 2.52	> 0.90
Other vascular surgery (N (%))	33 (4.8%)	8 (5.6%)	0.86	0.43, 1.96	0.70
Trauma (N (%))	20 (2.9%)	7 (4.9%)	0.59	0.27, 1.49	0.20
*Illness at RRT initiation*					
Time from ICU admission to RRT initiation (Hours)	142(380)	95(98)	47	14, 80	0.005
Cumulative Fluid Balance (mL/Kg)	51(65)	70(80)	-20	-34, -5.5	0.007
Urine Output (mL/kg)	754(1,001)	749(1,002)	4.2	-177, 186	> 0.90
Vasopressor support (N (%))	432 (63%)	83 (58%)	1.08	0.94, 1.27	0.30
SOFA score (points)	12.3(3.5)	11.8(3.7)	0.47	-0.19, 1.1	0.20
- Respiratory component	2.15(1.10)	2.13(1.10)	0.02	-0.18, 0.22	0.90
- Coagulation component	1.15(1.20)	1.15(1.13)	0.01	-0.20, 0.21	> 0.90
- Liver component	0.89(1.18)	0.80(1.14)	0.10	-0.11, 0.30	0.40
- Cardiovascular component	2.47(1.70)	2.20(1.75)	0.27	-0.04, 0.59	0.09
- Central nervous system component	2.42(1.53)	2.39(1.50)	0.03	-0.25, 0.30	0.80
- Renal component	3.22(0.88)	3.17(0.86)	0.05	-0.11, 0.20	0.60
Mechanical ventilation (N (%))	578 (84%)	115 (80%)	1.05	0.97, 1.15	0.30
*Laboratory values at RRT initiation*					
Hemoglobin(g/dL)	9.29(1.81)	9.13(1.80)	0.15	-0.18, 0.48	0.40
White blood cell count (× 10^9^/L)	18(14)	18(21)	-0.50	-4.1, 3.1	0.80
Platelets (× 10^9^/L)	161(116)	157(121)	3.7	-18, 25	0.70
Serum bilirubin (mg/dL)	48(86)	43(71)	4.9	-9.2, 19	0.50
Arterial pH	7.30(0.10)	7.34(0.09)	-0.03	-0.05, -0.02	< 0.001
Serum sodium (mmol/L)	138(7)	136(7)	2.0	0.73, 3.2	0.002
Serum creatinine (mg/dL)	4.82(2.03)	4.92(2.37)	-0.10	-0.52, 0.32	0.60
Serum potassium (mmol/L)	4.61(0.80)	4.50(0.74)	0.11	-0.03, 0.24	0.12
Serum bicarbonate (mmol/L)	19.4(4.9)	19.9(4.7)	-0.49	-1.3, 0.36	0.30
Blood urea nitrogen (mg/dL)	31(26)	27(14)	4.7	-0.23, 9.5	0.06
*Outcomes*					
RRT required at day 90 (N (%))	30 (4.4%)	8 (5.6%)	0.78	0.38, 1.80	0.50
Death at 90 days (N (%))	373 (54%)	49 (34%)	1.58	1.27, 2.04	< 0.001
Death between 1 and 7 days	170 (25%)	12 (8.4%)	2.95	1.69, 5.15	< 0.001
Death between 8 and 90 days*	203 (39%)	37 (28%)	1.39	1.04, 1.86	0.02
ICU death (N (%))	295 (43%)	36 (25%)	1.71	1.29, 2.34	< 0.001

eGFR, estimated glomerular filtration rate; ICU, intensive care unit; RRT, renal replacement therapy; SOFA, sequential organ failure assessment

^*^Among survivors at day 7 (Group A: 517, Group B: 131). Data is presented in count (%) or mean (SD)

ICU and 90-day mortality were higher in patients in class A compared to class B (43% vs. 25%, *p* < 0.001; 54% vs. 34%, p < 0.001). Both early (day 0–7) and late (day 8–90) mortality was higher in class A compared to class B (25% vs. 8.4%, *p* < 0.001); and (39% vs. 28%, *p* = 0.02); respectively. After adjustments for baseline characteristics, membership in class B was associated with a lower risk of 90-day mortality compared to patients with a class A (OR 0.48 CI 0.32; 0.70 *p* < 0.001). There was no interaction between the observed association and fluid balance at initiation of RRT (*p* = 0.30) and adding the probability of membership in class A as an adjustment variable in the model yielded similar results (OR 0.30 CI 0.09; 0.94 *p* = 0.04) (Table S3 and Table S4, supplementary material). Additional sensitivity analyses were performed including 1) the addition of potentially confounding variables (time from ICU admission to RRT initiation, RRT modality; Table S5 of the supplementary material) and 2) imputing patients in the excluded outlier class in class A or B (Table S6 and S7 of the supplementary material). These analyses revealed consistent results.

In joint longitudinal models, the results also supported a lower hazard of 90-day mortality associated with class B (aHR: 0.59 CI 0.45; 0.80 *p* < 0.001) (Table S8), which remained statistically significant after allowing separate trajectories for each latent class in the longitudinal component of the model (aHR: 0.6 CI: 0.44; 0.82 *p* < 0.001).

### Associations between NUF/CFB within identified fluid balance trajectories

After excluding the outlier class, within the cohort composed of Class A and Class B (N = 830), we observed an association between higher CFB and 90-day mortality (aHR 1.03 CI 1.01; 1.05 *p* = 0.004 per 100 mL/kg). No association was found between NUF and 90-day mortality (aHR: 0.95 CI 0.84; 1.07 *p* = 0.36 per 100 mL/kg). Separate analysis of latent classes showed a significant association between CFB and 90-day mortality within class A (N = 687) (aHR: 1.03 CI 1.00; 1.04 *p* = 0.03 per 100 mL/kg) and a significant association between NUF and 90-day mortality within class B (N = 143) (aHR: 1.74 CI 1.03; 3.26 *p* = 0.04 per 100 mL/kg).

### Trajectories in the accelerated arm

We repeated the latent trajectory analysis in patients who were allocated to the accelerated arm of the trial (N = 1347). Similarly, a three-class model including an outlier class (N = 49) and two classes with trajectories similar to previously described class A (N = 934) and B (N = 334) were observed (Figure S4 of the supplementary material). The separation between classes was considered fair (entropy of 0.74), although inferior to what was observed when participants of the standard arm were studied (Table S9 of the supplementary material). No statistically significant difference in 90-day mortality was found between the two latent classes in this cohort (OR: 0.94 CI 0.72–1.22 *p* = 0.6).

## Discussion

In a cohort of critically ill patients with severe AKI who commenced RRT, we showed that NUF/CFB evolved over time following two main latent trajectories which associated with different patient characteristics and survival. Class A, the most frequent, revealed a trajectory of fluid accumulation and stable ultrafiltration during the week following RRT initiation while class B revealed an early phase from day 0 to day 4 characterized by high NUF rate and decreasing CFB followed by a stabilization. At the end of the period, the NUF rate appeared similar in class B compared to class A.

Our work adds to the literature surrounding the increasingly complex epidemiology of fluid management related to RRT. Cohort studies have shown that fluid accumulation at the start of RRT and its persistence after initiation was associated with adverse outcomes in critically ill patients with AKI [2, 3]. A secondary analysis of the RENAL trial also reported that the achievement of a negative fluid balance early after initiation of RRT was generally associated with a better prognosis [5]. We report two common trajectories of fluid management in this specific setting with different characteristics, outcomes, and association with fluid related parameters.

Multiple recent cohort studies have reported an association between a high NUF (> 1.75 mL/kg/h) on continuous RRT early after initiation (≤ 48 h) and adverse outcomes [11, 20]. However, in our study, patients in class B—who achieved higher NUF rates early on and greater negative fluid balance—had improved outcomes compared to class A. This raises the question whether a more negative fluid balance achieved through intensified ultrafiltration causally leads to better outcomes (physician-driven hypothesis) or whether patients capable of tolerating higher ultrafiltration rates without hemodynamic compromise were inherently less severely ill (patient-driven hypothesis).

From a clinical practice standpoint, the reduction of substantial heterogeneity in NUF and CFB during the first week of RRT through classification into two broad fluid management trajectories may also reflect different practice patterns among clinicians. In recent international surveys [21, 22], clinicians reported competing priorities between promptly treating fluid accumulation and limiting the NUF rate to maintain hemodynamic stability and support organ recovery. Notably, there was no consensus regarding the recommended fluid removal rate (based on clinical vignettes), highlighting considerable variability between practitioners [22]. A recent study in sepsis found that variation in fluid administration attributable to physician practices was about four times greater than that explained by patient characteristics alone [23].

From a patients’ characteristics standpoint, although a greater proportion of patients with a class B trajectory were initially receiving CRRT, they had lower vasopressor use and a lower incidence of recent sepsis—features consistent with a population more likely to tolerate fluid removal due to physiological differences. For example, inflammation-related glycocalyx injury [24] and other factors associated with microvascular dysfunction may have been more prevalent in class A, contributing to fluid redistribution toward the extravascular space [25], impaired vascular refilling, and increased risk associated with rapid NUF. Consequently, despite our efforts to adjust statistically for baseline differences, it remains possible that the association between mortality and group A membership reflects underlying physiological processes that limit fluid removal during the first days of RRT, rather than effects of a specific fluid management strategy.

This study has significant limitations. First, the data is rooted in the STARRT-AKI trial in which participants were included only if the attending care team believed that immediate initiation of RRT was not required. Consequently, the studied cohort likely differs from an unselected cohort of critically ill patients, some of whom may need prompt RRT early during the course of AKI. Though similar classes were found in the accelerated arm of the trial, the association with 90-day mortality was not observed among participants randomized to this arm of the trial. This should be interpreted with caution since fluid management considerations may not have been a clinical priority. The presence of the latent classes identified in this study should be further validated in large cohorts of critically ill patients with AKI to determine if they are generalizable. Secondly, the data available for analysis does not contain information about physiological markers of tissue perfusion, fluid responsiveness and fluid tolerance, such as serum lactate, point-of-care ultrasound findings and response to passive-leg raising. Furthermore, the fluid removal goals of the attending care team were not collected. It is thus difficult to precisely determine whether the observed trajectories were due to fluid management decisions or related physiological factors such as tolerance to fluid removal. Finally, although hypothesis generating, these results should not be used to support a particular fluid management strategy. As with all observational studies, it is likely that residual confounding affected the association between the identified classes and outcomes.

## Conclusions

Two distinct fluid balance trajectories after initiation of RRT were identified through latent class trajectory analysis in the STARRT-AKI trial: a trajectory of early intensified ultrafiltration with negative fluid balance and a trajectory of stable fluid balance. Future studies should better characterize these sub-groups and clarify whether deliberate reduction in CFB through higher NUF mediates improved outcomes.

## Supplementary Information

Supplementary file 1

## Data Availability

The datasets used and/or analysed during the current study are available from the corresponding author on reasonable request provided a data sharing agreement is agreed upon.

## Abbreviations

AKI: Acute kidney injury
CFB: Cumulative fluid balance
ICU: Intensive care unit
NUF: Net ultrafiltration rate
RRT: Renal replacement therapy
