# Supplementary material for: Trajectories of fluid management after the initiation of renal replacement therapy in critically ill patients: a secondary analysis of the STARRT-AKI trial
Source: Crit Care. 2025 May 27;29:216. doi: 10.1186/s13054-025-05447-y (PMC12107945; doi:10.1186/s13054-025-05447-y)
Supplement: Supplementary file 1 [file 13054_2025_5447_MOESM1_ESM.pdf]

Supplementary material:

Supplementary methods:

The SOFA score was collected during the trial according to the chart below:

**System/points**	**0**	**1**	**2**	**3**	**4**	**Details**
**Respiration***	$PaO_2/FiO_2 \geq 400$	$PaO_2/FiO_2 < 400$	$PaO_2/FiO_2 < 300$	$PaO_2/FiO_2 < 200$ and ventilated	$PaO_2/FiO_2 < 100$ and ventilated	Lowest during the day
**Coagulation**	Platelets ≥ 150 $\times 10^9$/L	Platelets < 150 $\times 10^9$/L	Platelets < 100 $\times 10^9$/L	Platelets < 50 $\times 10^9$/L	Platelets < 20 $\times 10^9$/L	Lowest during the day
**Liver**	Bilirubin < 20 µmol/L	Bilirubin 20–32 µmol/L	Bilirubin 33–101 µmol/L	Bilirubin 102–204 µmol/L	Bilirubin > 204 µmol/L	Highest during the day
**Cardiovascular**	MAP ≥ 70 mmHg	MAP < 70 mmHg	Dopamine ≤ 5 µg/kg/min or any dose of dobutamine or milrinone	Dopamine 5.1-14.9 µg/kg/min or epinephrine/norepinephrine ≤ 0.1<br><br>µg/kg/min<br>or vasopressin ≤ 0.03 U/min or phenylephrine	Dopamine > 15 µg/kg/min or epinephrine/norepinephrine > 0.1 µg/kg/min<br>or vasopressin > 0.03 U/min or receipt of A-V extracorporeal life support	Highest during the day
**Central nervous system**	Glasgow coma scale (GCS) : 15	GCS 13–14	GCS 10–12	GCS 6–9	GCS < 6	Lowest during the day Patient's score should be based on actual abilities.
**Renal**	Creatinine < 110 µmol/L	Creatinine 110–170 µmol/L	Creatinine 171–299 µmol/L	Creatinine 300–440 or urine < 500 mL	Creatinine > 440 or urine < 200 mL	Highest during the day

* In the absence of arterial PaO_2 converstion tables from FiO2 were used from AARC Clinical Practice Guidelines. In Vitro pH and Blood Gas Analysis and Hemoximetry, Repiratory Care, 38:505-510, 1993.

Figure S1: Inclusion flowchart

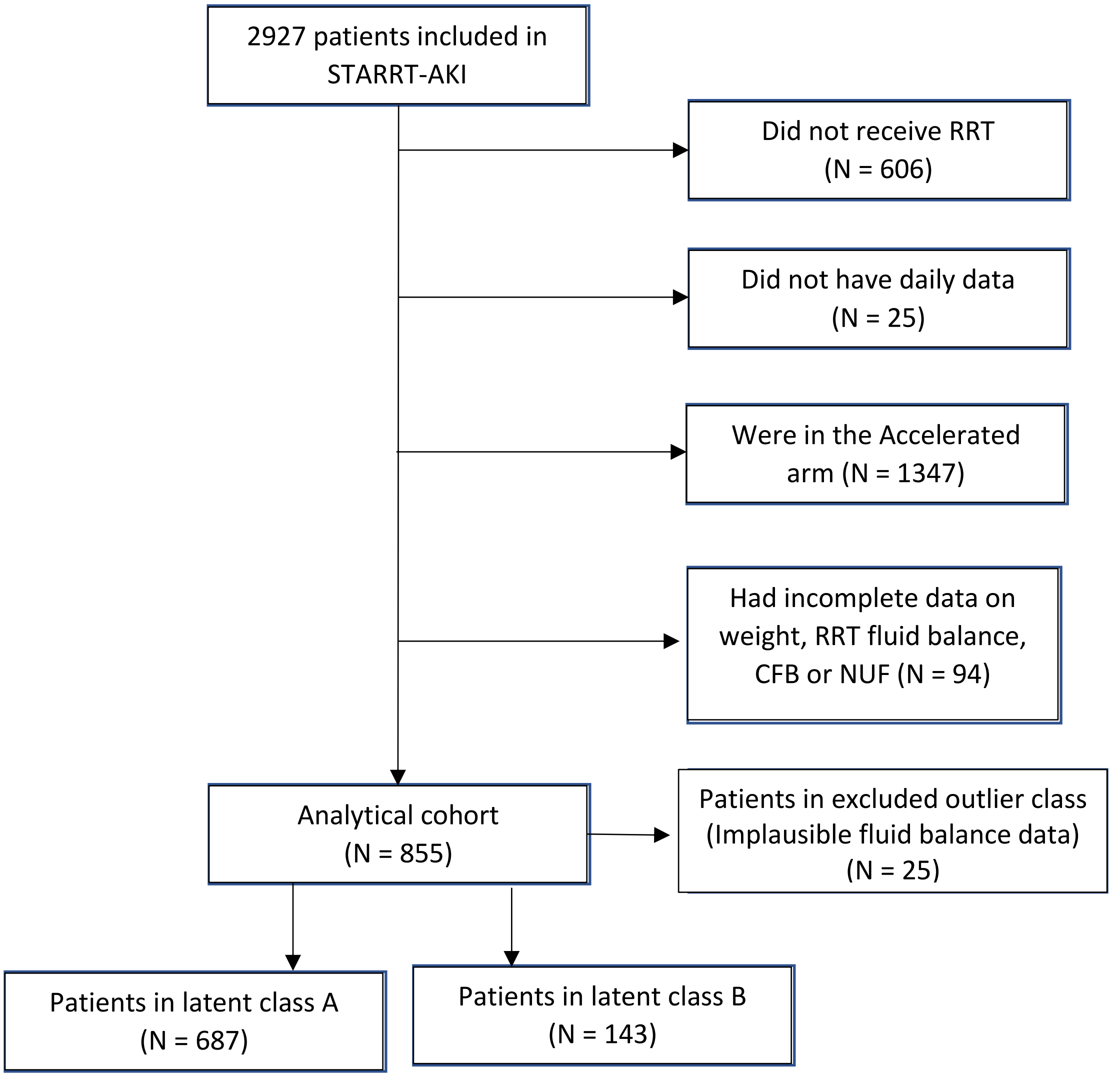

Figure S2: Estimated hazard ratio for mortality in relationship with time in days after RRT initiation. A) for cumulative fluid balance after renal replacement therapy initiation and for B) daily net ultrafiltration.

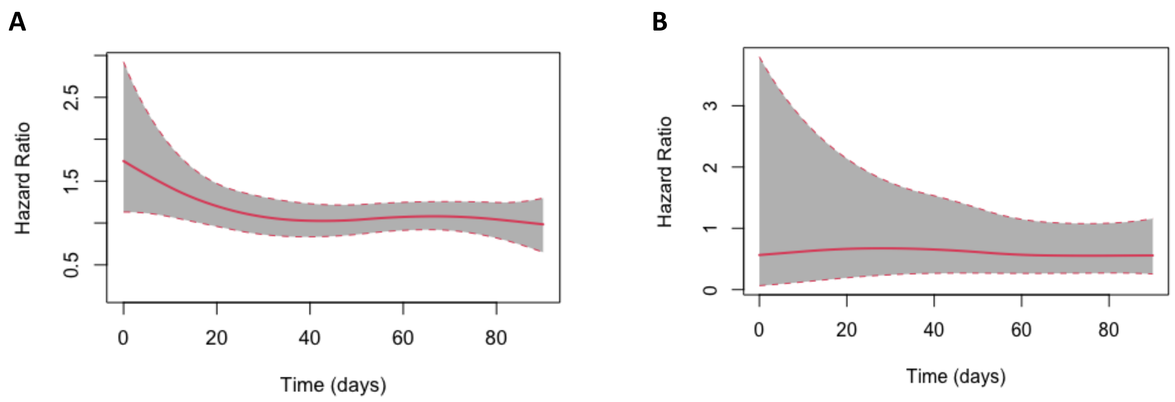

Figure S3: Trajectories of cumulative fluid balance (CFB) in mL/kg of body weight for the 3-class model. Class C (N=25) indicate implausible CFB data which likely is the result of incorrect data collection.

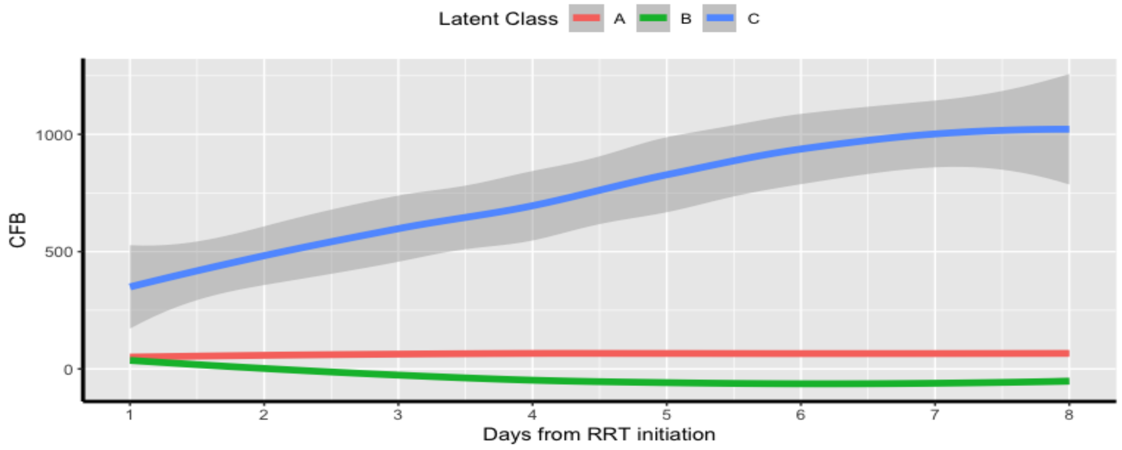

Figure S4: Predominant trajectories of A) cumulative fluid balance (CFB) in mL/kg and B) daily ultrafiltration rate (UF) in mL/kg/d identified using latent class analysis in the accelerated arm of the STARRT-AKI trial (Class A: 964 patients, Class B: 334 patients)

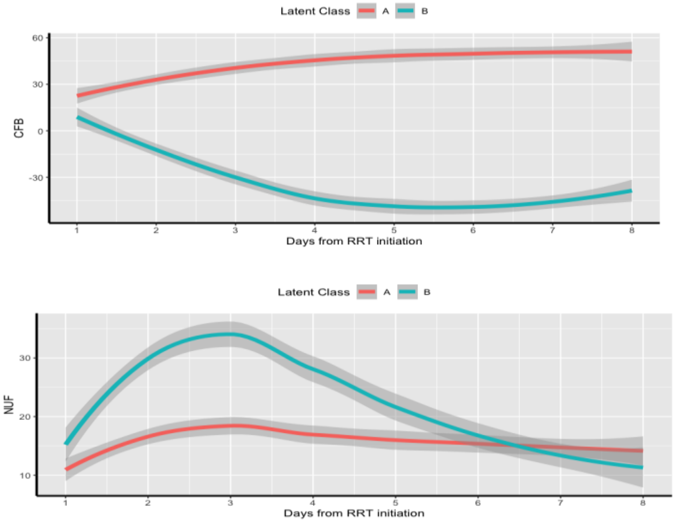

Table S1: Fluid balance data after initiation of renal replacement therapy in the standard arm of the STARRT-AKI trial.

**Characteristic**	**Day 0** N = 855	**Day 1** N = 822	**Day 2,** N = 771	**Day 3** N = 718	**Day 4** N = 657	**Day 5** N = 608	**Day 6** N = 557	**Day 7** N = 517
Modality								
Not on RRT	0 (0 %)	119 (14.48%)	140 (18.16%)	217 (30.22%)	252 (38.36%)	230 (37.83%)	230 (41.29%)	217 (41.97%)
CRRT	602 (70.41%)	565 (68.73%)	496 (64.33%)	392 (54.60%)	301 (45.81%)	274 (45.07%)	227 (40.75%)	198 (38.30%)
IHD	218 (25.50%)	114 (13.87%)	113 (14.66%)	90 (12.53%)	87 (13.24%)	86 (14.14%)	84 (15.08%)	85 (16.44%)
PIRRT	35 (4.09%)	24 (2.92%)	22 (2.85%)	19 (2.65%)	17 (2.59%)	18 (2.96%)	16 (2.87%)	17 (3.29%)
Ultrafiltration achieved - (mL/Kg of BW)	10.13 [22.81]	19.19 [39.55]	18.87 [37.95]	12.76 [34.57]	9.06 [33.28]	9.34 [31.81]	4.63 [33.31]	1.88 [27.86]
Cumulative fluid balance - (mL/Kg of BW)	56.73(95.37)	59.99(118.83)	61.73(144.36)	61.17(160.06)	60.71(183.54)	60.82(211.11)	61.68(217.93)	68.29(240.27)

Data is presented in n (%); Mean(SD) or Median [IQR]. Abbreviations: BW: Body weight, CRRT: Continuous renal replacement therapy, RRT: renal replacement therapy, IDH. Intermittent hemodialysis, PIRRT: Prolonged intermittent renal preplacement therapy.

Table S2: Patients characteristics of the outlier class compared to class A and class B.

Characteristic	Outlier class<br>N = 25	Class A<br>N = 687	Class B<br>N = 143
Age (Years)	64(12)	65(13)	62(15)
Body weight (Kg)	85(26)	89(24)	79(19)
Baseline GFR (mL/kg/1.73m2)	59(25)	67(30)	70(32)
Time from ICU admission to RRT initiation (Hours)	119(136)	142(380)	95(98)
SOFA score	13 (3.3)	12.3(3.5)	11.8(3.7)
Respiratory	2.72(1.02)	2.15(1.10)	2.13(1.10)
Coagulation	0.88(1.09)	1.15(1.20)	1.15(1.13)
Liver	0.56(1.12)	0.89(1.18)	0.80(1.14)
Cardiovascular	2.84(1.68)	2.47(1.70)	2.20(1.75)
Central nervous system	2.52(1.61)	2.42(1.53)	2.39(1.50)
Renal	3.52(0.65)	3.22(0.88)	3.17(0.86)
Urine Output (mL/kg)	537(804)	754(1,001)	749(1,002)
Cumulative Fluid Balance (mL/Kg)	146(123)	51(65)	70(80)
Hemoglobin(g/dL)	9.48(1.67)	9.29(1.81)	9.13(1.80)
White blood cell count	17(10)	18(14)	18(21)
Platelets	159(97)	161(116)	157(121)
Serum bilirubin (mg/dL)	37(93)	48(86)	43(71)
Arterial pH	7.30(0.11)	7.30(0.10)	7.34(0.09)
Serum sodium (mmol/L)	137(7)	138(7)	136(7)
Serum creatinine (mg/dL)	5.25(2.17)	4.82(2.03)	4.92(2.37)
Serum potassium (mmol/L)	4.49(0.65)	4.61(0.80)	4.50(0.74)
Serum bicarbonate (mmol/L)	19.0(4.4)	19.4(4.9)	19.9(4.7)
Blood urea nitrogen (mg/dL)	24(6)	31(26)	27(14)
Known chronic kidney disease (N (%))	13 (52%)	296 (43%)	57 (40%)
Known hypertension (N (%))	15 (60%)	390 (57%)	69 (48%)
Known diabetes (N (%))	8 (32%)	218 (32%)	45 (31%)
Known heart failure (N (%))	6 (24%)	90 (13%)	16 (11%)
Cardiopulmonary bypass (N (%))	0 (0%)	50 (7.3%)	16 (11%)
Aortic aneurysm repair (N (%))	3 (12%)	35 (5.1%)	7 (4.9%)
Other vascular surgery (N (%))	2 (8.0%)	33 (4.8%)	8 (5.6%)
Trauma (N (%))	0 (0%)	20 (2.9%)	7 (4.9%)
Sepsis in the last 72 hours (N (%))	15 (60%)	430 (63%)	70 (49%)
Mechanical ventilation (N (%))	20 (80%)	578 (84%)	115 (80%)
RRT required at day 90	2 (8.0%)	30 (4.4%)	8 (5.6%)
Death at 90 days	14 (56%)	373 (54%)	49 (34%)
- Death between 1 and 7 days	6 (24%)	170 (25%)	12 (8.4%)
- Death between 8 and 90 days*	9 (47%)	203 (39%)	37 (28%)
ICU death	11 (44%)	295 (43%)	36 (25%)

Legend: Data is presented in count (%) or mean (SD). Abbreviations: eGFR: estimated glomerular filtration rate, ICU: Intensive care unit, RRT: Renal replacement therapy, SOFA: Sequential Organ Failure Assessment.

Table S3: Association between UF/CFB trajectories and 90-day mortality.

Characteristic	OR	95% CI	p-value
**Latent Class**			
**Class A**	Ref.	—	—
**Class B**	0.48	0.32, 0.7	<0.001
**Age**	1.50	1.29, 1.75	<0.001
**Diabetes**	0.99	0.72, 1.35	>0.9
**Heart Failure**	1.32	0.85, 2.07	0.2
**Chronic kidney disease**	1.00	0.74, 1.35	>0.9
**Sepsis**	1.42	1.06, 1.92	0.02
**Cardiopulmonary bypass**	0.39	0.21, 0.68	0.001
**CFB at the initiation of RRT***	3.18	0.37, 27.8	0.3

Legend: Multivariable logistic regression models with the latent class membership defined as the predictor of interest for 90-day mortality. * No interaction was present between Latent class and CFB at the initiation of RRT (p=0.2).

Tables S4: Sensitivity analysis regarding the association between latent class membership and 90-day mortality with the probability of membership to class A included as an adjustment variable.

Characteristic	OR	95% CI	p-value
**Latent Class**			
**Class A**	Ref.	—	—
**Class B**	0.30	0.09, 0.94	0.04
**Probability of membership to class A**	0.57	0.15; 2.9	0.4
**Age (per year)**	1.50	1.29, 1.75	<0.001
**Diabetes**	0.99	0.72, 1.35	>0.9
**Heart Failure**	1.33	0.84, 2.07	0.2
**Chronic kidney disease**	1.00	0.74, 1.35	>0.9
**Sepsis**	1.42	1.06, 1.92	0.020
**Cardiopulmonary bypass**	0.38	0.21, 0.67	0.001
**CFB at the initiation of RRT (per L?)**	3.20	0.38, 27.9	0.3

Tables S5: Sensitivity analysis regarding the association between latent class membership and 90-day mortality with additional adjustment variables: Use of IHD/SLED as initial modality and Time from ICU admission to RRT initiation.

Characteristic	OR	95% CI	p-value
**Latent Class**			
**Class A**	Ref.	—	—
**Class B**	0.44	0.29, 0.65	<0.001
**Age (per year)**	1.51	1.30, 1.77	<0.001
**Diabetes**	0.96	0.70, 1.32	0.8
**Heart Failure**	1.37	0.88, 2.16	0.2
**Chronic kidney disease**	1.02	0.76, 1.38	0.9
**Sepsis**	1.45	1.07, 1.96	0.015
**Cardiopulmonary bypass**	0.37	0.21, 0.66	<0.001
**CFB at the initiation of RRT (per L?)**	5.11	0.58, 46.1	0.14
**Use of IHD/SLED as initial modality**	0.63	0.46, 0.87	0.005
**Time from ICU admission to RRT**	1.00	1.00, 1.00	0.5

Table S6: Sensitivity analysis regarding the association between latent class membership and 90-day mortality with patients from the outlier class (N=25) imputed as class A trajectory.

Characteristic	OR	95% CI	p-value
**Latent Class**			
**Class A**	Ref.	—	—
**Class B**	0.44	0.29, 0.65	<0.001
**Age (per year)**	1.52	1.31, 1.77	<0.001
**Diabetes**	0.92	0.67, 1.25	0.6
**Heart Failure**	1.46	0.95, 2.27	0.088
**Chronic kidney disease**	0.99	0.74, 1.33	>0.9
**Sepsis**	1.47	1.10, 1.98	0.010
**Cardiopulmonary bypass**	0.37	0.21, 0.66	<0.001
**CFB at the initiation of RRT (per L?)**	4.63	0.60, 37.0	0.14
**Use of IHD/SLED as initial modality**	0.62	0.45, 0.84	0.003
**Time from ICU admission to RRT**	1.00	1.00, 1.00	0.5

Table S7: Sensitivity analysis regarding the association between latent class membership and 90-day mortality with patients from the outlier class (N=25) imputed as class B trajectory.

Characteristic	OR	95% CI	p-value
**Latent Class**			
**Class A**	Ref.	—	—
**Class B**	0.49	0.33, 0.70	<0.001
**Age (per year)**	1.52	1.30, 1.77	<0.001
**Diabetes**	0.92	0.67, 1.25	0.6
**Heart Failure**	1.50	0.97, 2.33	0.069
**Chronic kidney disease**	1.00	0.75, 1.35	>0.9
**Sepsis**	1.47	1.09, 1.98	0.011
**Cardiopulmonary bypass**	0.37	0.20, 0.64	<0.001
**CFB at the initiation of RRT (per L?)**	6.36	0.82, 51.6	0.079
**Use of IHD/SLED as initial modality**	0.62	0.45, 0.86	0.004
**Time from ICU admission to RRT**	1.00	1.00, 1.00	0.5

Table S8: Sensitivity analysis regarding the association between latent class membership and 90-day mortality while including cumulative fluid balance (CFB) and daily net ultrafiltration (NUF) as longitudinal components in the model.

Characteristic	OR	95% CI	p-value
**Latent Class**			
**Class A**	Ref.	—	—
**Class B**	0.59	0.45, 0.80	0.002
**Age**	1.32	1.19, 1.47	<0.001
**Diabetes**	0.99	0.80, 1.20	0.926
**Heart Failure**	1.14	0.86, 1.49	0.338
**Chronic kidney disease**	0.96	0.78, 1.18	0.728
**Sepsis**	1.28	1.08, 1.56	0.006

Characteristic	OR	95% CI	p-value
**Cardiopulmonary bypass**	0.48	0.28, 0.74	<0.001
**CFB at the initiation of RRT**	1.58	0.38, 6.12	0.3
**Association of CFB and survival**	1.03	1.01, 1.05	0.004
**Association of NUF and survival**	0.95	0.84, 1.07	0.364

Table S9: Model evaluation criteria according to the number of latent classes in the accelerated arm participants.

	Bayesian information criteria (BIC)	Entropy	% class 1	% class 2	% class 3	% class 4
**1 Class**	95086.06	1.000	100%			
**2 Class**	96110.06	0.521	63.1%	36.9%		
**3 Class**	96367.16	0.737	1.3%	63.0%	36.9%	
**4 Class**	-96527.78	0.714	18.3%	66.3%	14.5%	0.9%
